# Quality of life in women with urinary incontinence seeking care using e-health

**DOI:** 10.1186/s12905-021-01477-0

**Published:** 2021-09-20

**Authors:** Ylva Åström, Ina Asklund, Anna Lindam, Malin Sjöström

**Affiliations:** 1 Department of Public Health and Clinical Medicine, Umeå University, 905 81 Umeå, Sweden; 2 Department of Public Health and Clinical Medicine, Unit of Research, Education and Development – Östersund, Umeå University, 905 81 Umeå, Sweden

**Keywords:** E-health, ICIQ-LUTSqol, ICIQ-UI SF, Quality of life, Urinary incontinence, Women

## Abstract

**Background:**

Quality of life (QoL) in women with urinary incontinence (UI) is mainly affected by UI severity, but it is also affected by the UI subtype, comorbidities, age, and socioeconomic status. e-Health is a new method for providing UI treatment. This study aimed to identify factors with the highest impact on QoL in women that turned to e-health for UI self-management.

**Methods:**

We analysed data from three randomized controlled trials (RCTs) that evaluated e-health treatments for UI. We included baseline data for 373 women with stress urinary incontinence (SUI) and 123 women with urgency/mixed UI (UUI/MUI). All participants were recruited online, with no face-to-face contact. Participants completed two questionnaires: the International Consultation on Incontinence Questionnaire-Urinary Incontinence Short Form (ICIQ-UI SF, range: 0–21 points), for assessing UI severity, and the ICIQ Lower Urinary Tract Symptoms Quality of Life (ICIQ-LUTSqol, range: 19–76 points), for assessing condition-specific quality of life (QoL). To identify factors that impacted QoL, we constructed a linear regression model.

**Results:**

The mean ICIQ-LUTSqol score was 34.9 (SD 7.6). UI severity significantly affected QoL; the adjusted mean ICIQ-LUTSqol score increased by 1.5 points for each 1.0-point increase in the overall ICIQ-UI SF score (*p* < 0.001). The UI type also significantly affected QoL; the adjusted mean ICIQ-LUTSqol score was 2.5 points higher in women with UUI/MUI compared to those with SUI (*p* < 0.001).

**Conclusions:**

We found that women that turned to e-health for UI self-management advice had a reduced QoL, as shown previously among women seeking UI care through conventional avenues, and that the severity of leakage had a greater impact on QoL than the type of UI. Condition-specific factors impacted the QoL slightly less among women that turned to e-health, compared to women that sought help in ordinary care. Thus, e-health might have reached a new group of women in need of UI treatment.

## Background

Urinary incontinence (UI) is common among women. It is defined as any involuntary leakage of urine [1], and it affects about 25% of adult women. There are three types of UI: stress urinary incontinence (SUI), urgency urinary incontinence (UUI), and mixed urinary incontinence (MUI) [2]. SUI is leakage that occurs with physical activity, sneezing, or coughing; UUI is leakage associated with, or immediately preceded by, a sudden need to void; and MUI is a combination of the symptoms of SUI and UUI [1]. Among women with incontinence, about one in ten experiences urine leaks every day. Both the prevalence and severity of UI increase with age [2]. SUI is the most common type. Among women with UI, about 50% have SUI. SUI is most common among the middle-aged, and thereafter, incidence decreases with age. In contrast, the incidences of UUI and MUI are higher among older women, and the incidences increase with age [3].

The first-line treatment for all types of UI is pelvic floor muscle training (PFMT). About two in three women achieve a cure or improvement with PFMT [4]. For UUI and MUI, bladder training can complement the PFMT treatment [5]. Before treatment, the type and severity of UI can be diagnosed based on patient-reported responses to questionnaires, voiding diaries, and validated rating scales [6].

Quality of life (QoL) is the recommended outcome measure for evaluating UI treatments [7]. QoL can be measured with various methods, including generic and condition-specific questionnaires. Previous studies have shown that women with UI have lower estimated QoL than women without UI, and QoL decreases with UI severity. Moreover, the type of UI affects QoL to different degrees; the UUI subtype has the highest negative impact on QoL, but other factors—such as age, socioeconomic parameters, concurrent medical conditions, and the duration of UI symptoms—have negative impacts on condition-specific QoL [8]. Nevertheless, only a minority of women with UI seek care [9]. The reasons for not seeking help include low expectations of treatment efficacy and embarrassment, due to the social stigma regarding UI [10, 11]. In addition, many women believe that UI is inevitable with age, should be accepted as normal, and that nothing can be done to improve the symptoms. Furthermore, some women distrust healthcare, in general [11]. Therefore, new, easily accessible ways of providing treatment are needed.

E-health is defined by the World Health Organization as the use of information and communication technologies for health. It is a growing field that may increase access to healthcare for those who, for example, have limited access to ordinary care or are unwilling or reluctant to seek care [12]. Through e-health, both diagnostics and training programmes for UI self-management can be provided without face-to-face contact [6, 13, 14].

Studies have shown that women that seek care through e-health were younger, had a higher level of education, and had a higher income than the general population [15]. It remains unknown whether the impact of UI on QoL is the same for women that seek UI care through e-health as for women that seek UI care through other avenues.

The purpose of this project was to evaluate condition-specific QoL among women that turned to e-health for UI self-management. We investigated how the severity of UI impacted QoL among women in this population. We investigated whether the QoL might be affected by the type of UI, age, education, and comorbidity, and whether UUI/MUI was associated with a lower QoL than SUI.

## Methods

### The eContinence research project

For this project, we retrieved the baseline data from three different randomized controlled trials (RCTs) performed in the eContinence research project at Umeå University, Sweden. These trials are registered on clinicaltrials.gov (IDs: NCT01032265, NCT01848938, NCT03097549), and altogether, they included 496 women. The eContinence project aimed to develop, evaluate, and implement treatment programmes for UI through e-health [16].

The three RCTs were conducted between 2009 and 2018. The aims were to evaluate SUI self-management via the internet (RCT one); to evaluate SUI self-management via the app, Tät® (RCT two); and to evaluate MUI and UUI self-management via the app, Tät® II (RCT three). Community-dwelling women were recruited from all over Sweden, via the project’s website [16]. For all three RCTs, there was no face-to-face contact between the study participants, researchers, and healthcare providers throughout the recruitment and treatment processes. Treatment effectiveness was evaluated in the short term (three months) [17–19] and over the long term (up to two years) [20, 21]. Previous studies found that UI treatments that focused mainly on PFMT, provided via the internet or a mobile app, showed clinically relevant improvements in symptoms and in QoL [17–21].

The enrolment process and the inclusion and exclusion criteria for the three RCTs are shown In Table 1. More detailed data were published previously [17–19].

**Table 1:** Overall information, inclusion and exclusion criteria, and enrolment process, in three randomized studies for women with urinary incontinence (UI) using e-health

	RCT one	RCT two	RCT three
Time span	2009–2011	2013–2014	2017–2018
Inclusion criteria	Female Ability to read and write Swedish Access to internet/smartphone/e-mail		
	Age 18–70 years SUI ≥ 1 episode/week	Age ≥ 18 years SUI ≥ 1 episode/week for the last 6 months	Age ≥ 18 years UUI/MUI ≥ 2 episode/week for the last 12 months
Exclusion criteria	Pregnancy Previous UI surgery Macroscopic haematuria Known malignancy in the lower abdomen Difficulties with passing urine lePara>Intermenstrual bleedings Impaired mobility or sensibility in the legs or lower abdomen		
	Severe psychiatric disorders, or HADS score > 15 for depression or anxiety Max. voiding volume < ≈ 0.3 L and mean micturition volume < ≈ 0.2 L	Severe psychiatric disorders Max. voiding volume < 0.3 L	Use of another PFMT app Use of mirabegron or antimuscarinic drugs Painful urges or micturition Previous pyelonephritis ≥3 urinary tract infections in the last 12 months Previous stroke, neurological disease, or diabetes. Max. voiding volume ≤ 0.15 L
Study invitations	www.econtinence.se		
	Daily newspapers Websites for medical advice	Daily newspapers On the web Facebook Posters at health centres and training centres	Newspapers Facebook Radio TV
Enrolment process	1. Screening questionnaire homepage (n = 684) 2. Postal questionnaire, informed consent, 2-day bladder diary (n = 287) 3. Telephone interview urotherapist (n = 277)	1. Screening questionnaire homepage (n = 805) 2. Informed consent, 2-day bladder diary, maximum voiding volume (n = 345) 3. Web-based questionnaire, telephone interview (n = 129)	1. Screening questionnaire homepage (n = 1241) 2. Informed consent, 2-day bladder diary, maximum voiding volume, web-based questionnaire, telephone interview incontinence nurse or general practitioner (n = 142)
Randomization	250 women	123 women	123 women
Age span	23–70 years	27–72 years	31–77 years
Randomization arms	Internet-based treatment programme (n = 124) Postal treatment programme (n = 126)	Treatment app (n = 62) Control group (n = 61)	Treatment app (n = 60) Information app (n = 63)

*RCT* Randomized controlled trial; *UI* Urinary incontinence; *SUI* Stress UI; *UUI/MUI* Urgency UI/Mixed UI; *HADS* Hospital Anxiety and Depression Scale; *PFMT* Pelvic Floor Muscle Training

### Questionnaires

The baseline data collected in the RCTs included age, education, use of prescription drugs, the severity of leakage, based on the International Consultation on Incontinence Questionnaire-Urinary Incontinence Short Form (ICIQ-UI SF) score [22], and the condition-specific QoL, based on the International Consultation on Incontinence Questionnaire Lower Urinary Tract Symptoms Quality of Life (ICIQ-LUTSqol) score [23].

The ICIQ-UI SF is a highly recommended [7], validated questionnaire for evaluating UI symptom severity [22]. It includes three items that participants rate: leakage frequency, amount of leakage, and overall impact on QoL. It also includes an unrated, self-diagnostic item for assessing the type of UI. The rating scale is additive, and scores range from 0 to 21, where greater values indicate worse severity. The overall scores can be divided into four severity categories: slight = 1–5 points, moderate = 6–12 points, severe = 13–18 points, and very severe = 19–21 points [24].

The ICIQ-LUTSqol is a highly recommended [7], validated questionnaire for evaluating condition-specific QoL [23]. It includes 19 items that are scored on a scale of 1–4 points, and the overall score ranges from 19 to 76, where greater values indicate a larger impact on the QoL. The ICIQ-LUTSqol items can be divided into six different domains, including role limitation, physical limitations, social limitations, personal relationships, emotions, and sleep [25].

We used the ICIQ questionnaires with permission from the ICIQ project office.

### Definitions

We defined comorbidity as the regular use of any prescribed medication (in RCTs one and two) or any prescribed medication for heart and vessel diseases, oestrogen, hormonal intrauterine device (IUD), or drugs for treating incontinence, depression, anxiety, or asthma (in RCT three).

We used the educational level (i.e., any vs. no university education) as a proxy for socio-economic status.

### Statistics

For this study, we analysed the baseline datasets previously collected in the three RCTs. For these RCTs, participants completed the ICIQ-UI SF and ICIQ-LUTSqol at baseline and at each follow-up. Among the baseline datasets, five missing items were found in RCT one, and one missing item was found in RCT two. Therefore, to calculate the overall baseline scores, we imputed the scores from the corresponding questionnaires completed at the first follow-up in each study. Values were only imputed when a single or a few responses were missing; when more responses were missing, the overall score was set as ‘missing’.

To compare groups, we performed the Chi-square test for categorical variables and the ANOVA or the independent-samples t-test for continuous variables. For non-normally distributed data, we performed the Kruskal-Wallis test or Fisher’s exact test, when the group sample size was small.

We constructed a linear regression model to evaluate the impact of age, university education, comorbidity, type of UI, and UI severity on the ICIQ-LUTSqol scores, and thereby, the impact on the condition-specific QoL. Both unadjusted and adjusted analyses were performed, and 95% confidence intervals (CI) were calculated. For the adjusted analyses, the model was adjusted for age, university education, comorbidity, type of UI, and the ICIQ-UI SF score. A significance level of 0.05 was used for all analyses. We performed all analyses with SPSS statistics version 27 (IBM, Armonk, NY, USA).

## Results

The baseline characteristics of all participants and of participants in each of the three RCTs are presented in Table 2. Women with UUI/MUI (RCT three) were older, had a higher BMI, and had more severe leakage, and higher impact on QoL, than the women with SUI (RCTs one and two). Participants with the highest level of education were found in RCT two, followed by RCT three.

**Table 2:** Baseline characteristics in women with urinary incontinence (UI) who have sought care using e-health, in three randomized studies

Variable	RCT one^c^ (n = 250)	RCT two^c^ (n = 123)	RCT three^d^ (n = 123)	*p* values	Total (n = 496)
Age, years, mean (SD)	48.6 (10.2)	44.7 (9.4)	58.3 (9.5)	< 0.001^ h^	50.1 (11.0)
BMI, kg/m^2^, median (IQR)	23.5 (21.9–26.5)^a^	23.2 (21.4–25.9)	25.2 (23.2–28.6)	< 0.001^i^	23.9 (22.0-26.9)^a^
University education ≥ 3 years, n (%)	135 (54.0)	98 (79.7)	79 (64.2)	< 0.001^j^	312 (62.9)
Any university education, n (%)	188 (75.2)	107 (87.0)	105 (85.4)	0.008^j^	400 (80.6)
Daily smokers, n (%)	9 (3.6)	5 (4.1)	0 (0)	0.055^k^	14 (2.8)
Nulliparous, n (%)	16 (6.4)	9 (7.3)	15 (12.2)	0.145^j^	40 (8.1)
Comorbidity^e^, n (%)	102 (41.1)^b^	52 (42.3)	43 (35.0)	0.428^j^	197 (39.9)^b^
ICIQ-UI SF score^f^, mean (SD)	10.4 (3.3)	11.1 (2.8)	11.6 (3.3)	0.003^ h^	10.9 (3.2)
Severity of UI:				0.095^k^	
Slight, n (%):	14 (5.6)	3 (2.4)	3 (2.4)		20 (4.0)
Moderate, n (%):	170 (68.0)	78 (63.4)	73 (59.3)		321 (64.7)
Severe/Very severe, n (%):	66 (26.4)	42 (34.1)	47 (38.2)		155 (31.3)
ICIQ-LUTSqol score^g^, mean (SD)	33.6 (7.5)^a^	34.4 (6.1)	37.8 (8.2)	< 0.001^ h^	34.9 (7.6)^a^

*RCT* randomized controlled trial, *BMI* Body Mass Index, *SD* standard deviation; *IQR* Interquartile range

^a^One missing

^b^Two missing

^c^Women with Stress UI

^d^Women with Urgency UI/Mixed UI

^e^Comorbidity defined as regularly use of prescription drugs

^f^International Consultation on Incontinence Questionnaire-Urinary Incontinence Short Form (ICIQ-UI SF) measuring symptoms

^g^International Consultation on Incontinence Questionnaire Lower Urinary Tract Symptoms Quality of Life (ICIQ-LUTSqol) measuring condition-specific quality of life

^h^*p* values by ANOVA

^i^*p* values by Kruskal-Wallis test

^j^*p* values by Chi-square test

^k^*p* values by Fisher’s exact test

UI severity and QoL are presented for each UI subtype group in Table 3. Compared to participants with SUI, those with UUI or MUI had more severe leakage (mean ICIQ-UI SF scores: 11.6, SD 3.3 vs. 10.6 SD 3.2), which had a higher impact on QoL (mean ICIQ-LUTSqol scores: 37.8, SD 8.2 vs. 33.9, SD 7.1).

**Table 3:** ICIQ-UI SF score, severity of UI and ICIQ-LUTSqol score for women with stress urinary incontinence (SUI) versus urgency or mixed urinary incontinence (UUI/MUI), who have sought care using e-health

Variables	SUI (n = 373)	UUI/MUI (n = 123)	*p* value
ICIQ-UI SF score, mean (SD)	10.6 (3.2)	11.6 (3.3)	0.004^b^
Severity of UI:			0.136^c^
Slight, n (%):	17 (4.6)	3 (2.4)	
Moderate, n (%):	248 (66.5)	73 (59.3)	
Severe/Very severe, n (%):	108 (29.0)	47 (38.2)	
ICIQ-LUTSqol score, mean (SD)	33.9 (7.1)^a^	37.8 (8.2)	< 0.001^b^

*ICIQ-UI SF* international consultation on incontinence questionnaire-urinary incontinence short form, *ICIQ-LUTSqol* international consultation on incontinence questionnaire lower urinary tract symptoms quality of life, *SD* standard deviation

^a^One missing

^b^*p* values by independent-samples *t*-test

^c^*p* value by Fisher’s exact test

**Fig. 1:**
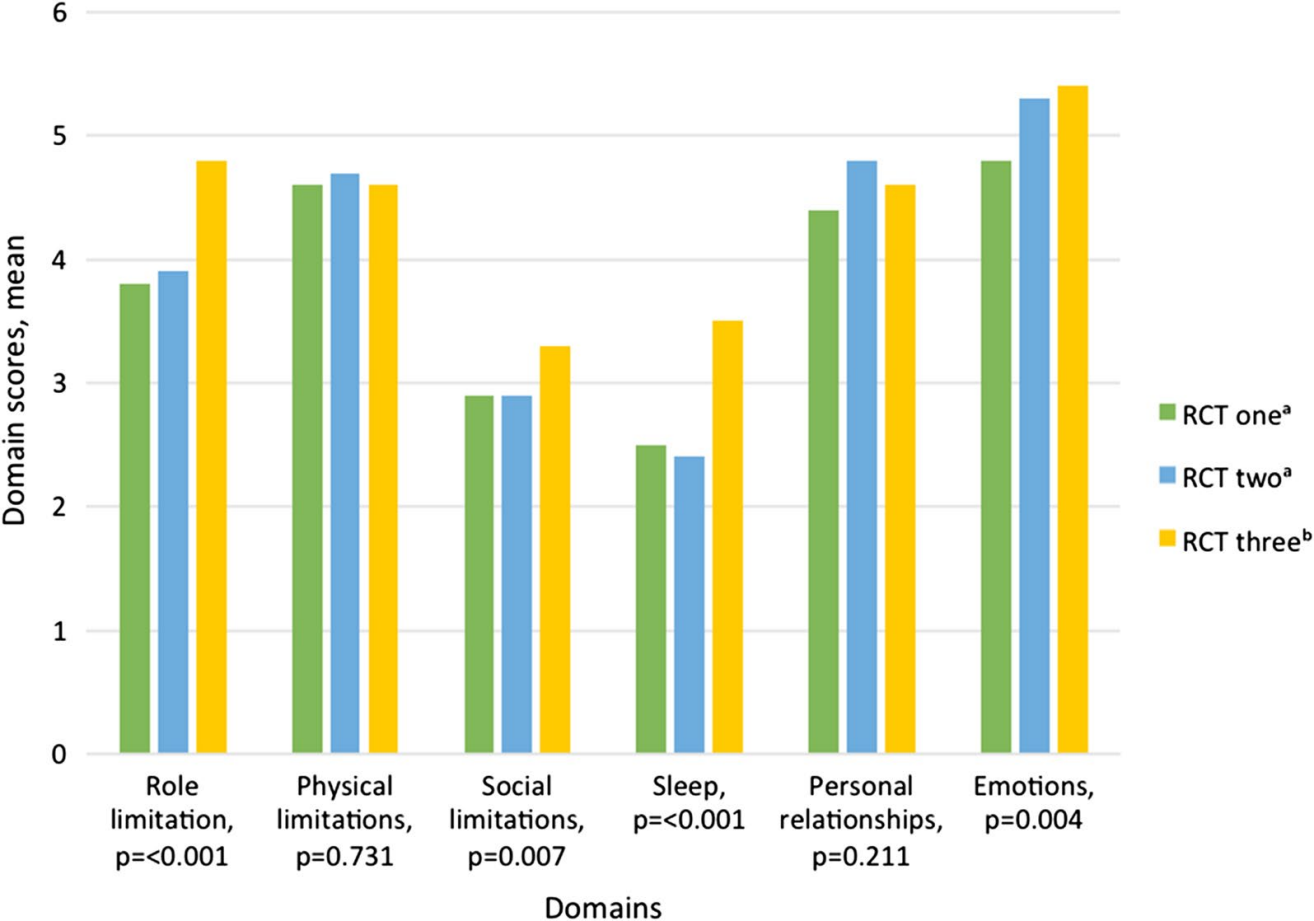
ICIQ-LUTSqol domain scores for women with urinary incontinence (UI), who have sought care using e-health, in three different randomized controlled studies (RCT). ^a^ Women with Stress UI; ^b^ Women with Urgency UI/Mixed UI. One missing in Personal relationships in RCT one. *p* values by ANOVA. 2–8 points: *Role limitation*: ICIQ-LUTSqol question 2a and 3a; *Physical limitations*: ICIQ-LUTSqol question 4a and 5a; *Social limitations*: ICIQ-LUTSqol question 6a and 7a; *Sleep*: ICIQ-LUTSqol question 14a and 15a. 3–12 points: *Personal relationships*: ICIQ-LUTSqol question 8a, 9a, and 10a; *Emotions*: ICIQ-LUTSqol question 11a, 12a, and 13a. *ICIQ-LUTSqol* International Consultation on Incontinence Questionnaire Lower Urinary Tract Symptoms Quality of Life

Overall, UI had the greatest impact on the QoL domains of physical limitations (mean score: 4.6, SD 1.2, 57.5% of maximum score), role limitations (mean score: 4.1, SD 1.4, 51.3% of maximum score), and emotions (mean score: 5.1, SD 1.8, 42.5% of maximum score). The impact of leakage on the different ICIQ-LUTSqol domains are presented for the three RCTs in Fig. 1. Among women with UUI/MUI, leakage had a significantly greater impact on the domains of role limitation, social limitations, emotions, and sleep, compared to women with SUI.

The linear regression model results are presented in Table 4. The adjusted results showed that increasing leakage severity was significantly associated with worsening QoL. Indeed, for each 1.0 point increase in the mean overall ICIQ-UI SF score, the mean overall ICIQ-LUTSqol score increased by 1.5 points. The type of UI also significantly affected the QoL; the impact of UUI/MUI increased by 2.5 points compared to the impact of SUI on the overall ICIQ-LUTSqol score. In addition, the mean ICIQ-LUTSqol score was on average 2.1 points higher among participants without any university education compared to those with some university education. Moreover, the mean ICIQ-LUTSqol score was on average 1.2 points higher among participants with comorbidities, compared to those without any comorbidity. Age showed no significant effect on the ICIQ-LUTSqol in the model.

**Table 4:** Regression analysis summary for factors impacting on ICIQ-LUTSqol scores, for women with UI, using e-health

Variable	Unadjusted B^a^	95% CI	*p* value	Adjusted B^a^	95% CI	*p* value
Age (n = 495) (continious variables 23–77)	0.1	0.0-0.1	0.038^c^	< 0.1	0.0–0.1	0.377^c^
Any university education (n = 495) (yes or no)	2.4	0.7–4.1	0.005^c^	2.1	0.9–3.4	0.001^c^
Comorbidity (n = 493) (no comorbidity compared to having comorbidity)	1.9	0.5–3.3	0.006^c^	1.2	0.2–2.2	0.023^c^
Type of UI (n = 495) (SUI compared to UUI/MUI)	3.9	2.4–5.4	< 0.001^c^	2.5	1.2–3.7	< 0.001^c^
ICIQ-UI SF score (n = 495) (continious variables 0–21)	1.6	1.4–1.7	< 0.001^c^	1.5	1.3–1.7	< 0.001^c^

Adjusted total no: 493. For the adjusted analyses, the model was adjusted for age, university education, comorbidity, type of UI, and the ICIQ-UI SF score

International Consultation on Incontinence Questionnaire Lower Urinary Tract Symptoms Quality of Life (ICIQ-LUTSqol) is a continious variable valued at 19–76

*UI* urinary incontinence, *SUI* stress UI, *UUI/MUI* urgency UI/mixed UI, *ICIQ-UI SF* international consultation on incontinence questionnaire-urinary incontinence short form, *CI* confidence interval

^a^B: regression coefficient

^b^Comorbidity defined as regularly use of prescription drugs

^c^*p* values by Linear regression

## Discussion

In this secondary analysis of baseline data from three RCTs, we found that QoL was reduced in women that sought care for UI through e-health, and that the severity of leakage had the greatest impact on QoL. The type of UI also affected QoL, but not to the same extent. The absence of a university education and the presence of comorbidity both had negative impacts on the QoL, but age alone had no significant effect.

In this study, the mean ICIQ-UI SF score for all included women was 10.9, which corresponded to moderate leakage [24], and the mean ICIQ-LUTSqol score was 34.9. Slightly lower scores were found in a survey conducted in 2015 in the UK, France, Germany, and the USA. That study included 1203 women 45–60 years old with unspecified subtypes of UI that completed a questionnaire via the internet. Those results showed moderate leakage (overall ICIQ-UI SF score 8.7) and a mean ICIQ-LUTSqol score of 32.8 [26]. Another RCT from urban parts of Malaysia studied women that sought care for UI through conventional avenues. Their baseline data on 120 women with SUI that received non-surgical treatment for UI showed a mean ICIQ-UI SF score of 10.0 and a mean ICIQ-LUTSqol score of 39.0. Thus, they observed a slightly lower UI severity, with a slightly higher impact on QoL, compared to our study population [27]. Another RCT, conducted in the UK, included 600 women that received new clinical diagnoses of SUI or MUI in centres that provided incontinence care. Compared to our study, they found a slightly higher mean ICIQ-UI SF score (12.4), but within the moderate severity range, and a somewhat higher mean ICIQ-LUTSqol score (42.9) [28]. Thus, although the level of severity was moderate in all these studies, the impact on QoL in our study population was slightly higher, compared to women in the internet survey, but slightly lower, compared to women that sought care for UI through conventional avenues. These results suggested that by the use of e-health, the eContinence project might have reached a new group of women that perhaps would not have sought UI care through conventional avenues, but had UI that clearly impacted their QoL.

Overall, among the participants in our study, UI had the highest impact on the QoL domains of physical limitations, role limitations (including household tasks and daily activities), and emotions. The women with UUI/MUI had more severe leakage and experienced a higher impact on social limitations, emotions, role limitations, and sleep, than the women with SUI. We have found no previous study that compared SUI to UUI/MUI and considered the ICIQ-LUTSqol domains. However, in a study by Abrams et al. (2015), the QoL domains were compared among participants divided into severity categories. They found that women with more severe UI experienced the greatest impact on QoL in the domains of social limitations and emotions [26].

Our regression analysis showed that, in our population, UI severity had the greatest impact on QoL. This finding was expected, based on previous studies. A large study conducted in 2007 on women that sought UI care through avenues other than e-health showed that severity was the single most important predictor of QoL among women with UI, regardless of the type of UI [8]. Another study conducted in 2018 explored relationships between mental health, sleep, and physical function and the UI type and severity. They showed that, among 510 women that sought help for UI symptoms, the severity, rather than the type of UI, had the greatest impact on anxiety, depression, and stress [29].

At first sight, our study results might appear to indicate that the UI type was the most important factor, based on the adjusted beta of 2.5; in contrast, UI severity only showed an adjusted beta of 1.5. However, it should be borne in mind that the UI type was a dichotomous variable; thus, there was only one step of comparison. In contrast, UI severity (according to ICIQ-UI SF) was a continuous variable that reflected many more steps of comparison; thus, UI severity had a much greater potential impact on the ICIQ-LUTSqol score.

### Strengths

To our knowledge, this study was the first to evaluate condition-specific QoL specifically in women with UI that sought care through e-health. One strength of this study was the relatively large number of participants and the small amount of missing data. Another strength was that the participants were actively seeking treatment, and thus, they represented a clinically relevant group. Moreover, the research group conducting the studies had solid clinical competence, and the diagnoses of SUI and UUI/MUI were well established. In the analyses, we were able to include many variables that could potentially affect QoL, and we worked in close collaboration with a statistician. For easier comparison with other studies, we used validated, recommended questionnaires to measure UI severity and condition-specific QoL [7, 22, 23]

### Limitations

This study also had some potential limitations. First, the UUI/MUI group had a considerably smaller number of participants than the SUI group (123 versus 373 women), and this might have affected the results. Additionally, 80.6% of participating women had a university education, compared to 47% of all Swedish women aged 25–64 years in 2015 [30]. Thus, the results from our population might not be generalizable to all women with UI in need of treatment. However, to date, e-health is mostly used by individuals with a higher education [15]; therefore, our population might have been representative of women that seek care through ehealth. Another limitation was that our data were restricted to data collected in previous RCTs. Thus, other factors that we did not investigate might also have influenced the QoL of our participants. For example, psychological illness might have an impact on condition-specific QoL, but questions about anxiety and depression were only included in the baseline questionnaires in two of the three RCTs; thus, they could not be further explored. Moreover, we might have underestimated the presence of some comorbidities (e.g., endocrinological diseases etc.), particularly in RCT three, due to the definition used. Our choice of definition was based on the fact that the three RCTs used different wording in the questions regarding prescribed medications and concurrent diseases. RCTs one and three had comparable data on prescription drugs and corresponding diseases; therefore, we used prescribed medications as a marker of comorbidity. Finally, eight years had passed from the start of the first RCT to the start of the third RCT. During that time period, the fast-growing field of e-health had developed rapidly, and this may have affected the results.

### Clinical implications and future perspectives

Our study showed that women with UUI/MUI and SUI that sought care through e-health experienced an impact on condition-specific QoL, mainly related to UI severity, rather than UI type. UI treatment can decrease symptom severity, and therefore, improve QoL; thus, it is important to provide effective, easily accessible treatments to everyone with UI, regardless of the subtype. Individual assessments of patients with UI are also needed, with careful assessments of the severity of leakage, to provide adequate help.

A considerable amount of research has been performed to investigate QoL among women with UI, in general, but not specifically among women that sought medical care for UI through e-health. Our study contributes new knowledge about this group of women, which may help to develop and improve treatments through e-health. Currently, the app, Tät® (RCT two) is freely available at the App Store and Google Play in several languages, including Swedish, English, Arabic, and Spanish. This app is intended for individuals that want to self-manage SUI, but it may also be useful as a complement to other treatments. We also aim to release the Tät ®II (RCT three) app to the public in the future. Treatments based on apps will not suit all women with UI, but they might contribute to new, cost-effective ways to help many women, and they may lead to an improvement in their QoL. Easily accessible self-management treatment programmes, through the internet or mobile applications, may facilitate access to medical care for this group of patients, and at the same time, relieve pressure on primary care.

Future research should investigate factors that separate this study population of e-health users from individuals that seek care through conventional avenues.

## Conclusions

Women that turned to e-health for self-management of UI had a reduced QoL. However, condition-specific factors impacted the QoL slightly less among women that turned to e-health, compared to women that sought help in ordinary care. These results suggested that e-health might reach a new group of women in need of UI treatment. Although QoL was affected more by UUI/MUI than by SUI, it was affected most by the severity of leakage in this population.

## Data Availability

The datasets used in the current study are available from the corresponding author on reasonable request.

## Abbreviations

BMI: Body Mass Index
CI: confidence interval
ICIQ-LUTSqol: international consultation on incontinence questionnaire lower urinary tract symptoms quality of life
ICIQ-UI SF: international consultation on incontinence questionnaire-urinary incontinence short form
MUI: mixed urinary incontinence
PFMT: pelvic floor muscle training
QoL: quality of life
RCT: randomized controlled trial
SD: standard deviation
SUI: stress urinary incontinence
UI: urinary incontinence
UUI: urgency urinary incontinence
